# Eruption cyst associated with right maxillary deciduous first molar

**DOI:** 10.11604/pamj.2018.30.285.15368

**Published:** 2018-08-23

**Authors:** Thorakkal Shamim, Koyakkunjakath Padinhare Ottayil Shabeer

**Affiliations:** 1 Department of Dentistry, Government Taluk Head Quarters Hospital, Malappuram, Kerala, India

**Keywords:** Eruption cyst, deciduous teeth, ruptured

## Image in medicine

**Figure 1:**
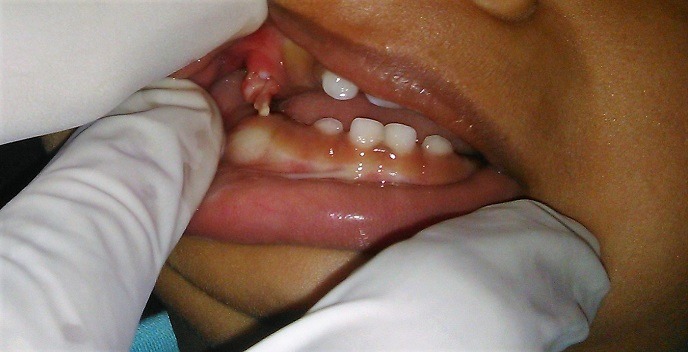
eruption cyst

A one and half-year-old boy presented to dental outpatient department with a soft tissue mass hanging from the right upper back milk tooth region for the past 2 days. The patient was asymptomatic and there was no fluid discharge. On clinical examination, it was found that the mass is friable with a size of 0.5 x 0.5cm and the roof of the mass was seen ruptured and hanging from the sides of the erupting right deciduous maxillary first molar (fig.1). A diagnosis of eruption cyst was made. Eruption cyst usually present as solitary swelling on the alveolar ridge mucosa. The treatment of the intact eruption cyst will be surgical excision and exposure of the unerupted primary teeth. In our case, eruption cyst is ruptured and wait and watch instruction was given and in the next follow-up, cyst was disappeared and the tooth was seen erupting normally.

